# High degree of polarization of the near-band-edge photoluminescence in ZnO nanowires

**DOI:** 10.1186/1556-276X-6-501

**Published:** 2011-08-19

**Authors:** Gwenole Jacopin, Lorenzo Rigutti, Andres De Luna Bugallo, François Henry Julien, Camilla Baratto, Elisabetta Comini, Matteo Ferroni, Maria Tchernycheva

**Affiliations:** 1 Institut d'Electronique Fondamentale, Université Paris Sud XI, UMR 8622 CNRS, 91405 Orsay, France; 2 CNR-IDASC SENSOR Lab., University of Brescia, Brescia, Italy

**Keywords:** zinc oxide, nanowire, photoluminescence, polarization

## Abstract

We investigated the polarization dependence of the near-band-edge photoluminescence in ZnO strain-free nanowires grown by vapor phase technique. The emission is polarized perpendicular to the nanowire axis with a large polarization ratio (as high as 0.84 at 4.2 K and 0.63 at 300 K). The observed polarization ratio is explained in terms of selection rules for excitonic transitions derived from the k·p theory for ZnO. The temperature dependence of the polarization ratio evidences a gradual activation of the *X*_C _excitonic transition.

PACS: 78.55.Cr, 77.22.Ej, 81.07.Gf.

## Introduction

One-dimensional nanoscale semiconductors have recently attracted considerable attention as promising candidates for innovative device applications. Their high surface to volume ratio can be exploited for the development of a new generation of chemical and biological sensors [1-3]. The wide bandgap (3.37 eV) of ZnO associated with its large exciton binding energy (60 meV) also makes it one of the most promising materials for photonic devices, such as light-emitting diodes [4] and lasers [5]. Thanks to the spatial separation of photogenerated carriers, UV photodetectors with a very high photoconductive gain based on ZnO nanowires (NWs) have been demonstrated [6]. It has been shown that the photodetection properties of ZnO NWs depend on the light polarization [7].

The photoluminescence of ZnO is typically composed of a near-band-edge (NBE) peak due to excitonic recombination and of a broad emission band in the visible range related to deep defect states [8-10]. The polarization properties of the luminescence of ZnO have been studied in bulk crystals [11-15]. However, these studies provided no theoretical explanation of the polarization behavior, especially of its temperature dependence. In the specific case of NWs, several studies have been carried out, but they were focused on the interpretation of the different behavior of defect and NBE luminescence [16,17]. As shown in other semiconductor NW systems [18], the polarization dependence in NWs results from two competitive phenomena: bulk crystal symmetry (imposing polarization perpendicular to *c*-axis) [19] and dielectric contrast in thin NWs (privileging polarization parallel to the NW axis) [7,20-23].

In this work, we have studied the polarization-resolved microphotoluminescence (μ-PL) of ZnO nanowires. We measured the polarization dependence of the NBE luminescence for temperature from 4.2 to 300 K. The experimental results are interpreted in the framework of the k·p model, allowing for the evaluation of the polarization ratio for each exciton type in bulk ZnO. The temperature dependence of the polarization ratio evidences a gradual activation of the *X*_C _excitonic transition.

## Experimental details

ZnO NWs are prepared by means of vapor transport process, in which the source material is vaporized and transported by a gas carrier towards the substrates where it condenses [24]. The experimental setup consists of a furnace capable to reach temperatures needed for oxide evaporation, a vacuum-sealed alumina tube connected to a vacuum pump, an automated valve, and a mass flow meter to control pressure and carrier flux. Adjusting the deposition conditions such as temperature of evaporation and carrier gas composition and flux, one-dimensional nanostructures can be obtained.

Platinum catalyst particles are firstly dispersed onto silicon substrates by DC magnetron sputtering at a working pressure of 5 × 10^-3 ^mbar and 50 W applied power. The source material is positioned at the middle of the alumina tube and evaporated at a temperature of 1,370°C at a pressure of 100 mbar. The platinum catalyzed substrates are placed onto an alumina holder and positioned inside the tube in an area corresponding to a temperature *T *= 660°C. Furnace heating from room temperature to 1,370°C lasts 1.5 h. During furnace heating and cooling, a reverse Ar gas flow (from the substrates to the powder) is applied to avoid uncontrolled mass deposition under transient conditions. Once the desired temperature is reached, the deposition conditions are kept for 15 min, and afterwards, the furnace is cooled down to room temperature.

**Figure 1:**
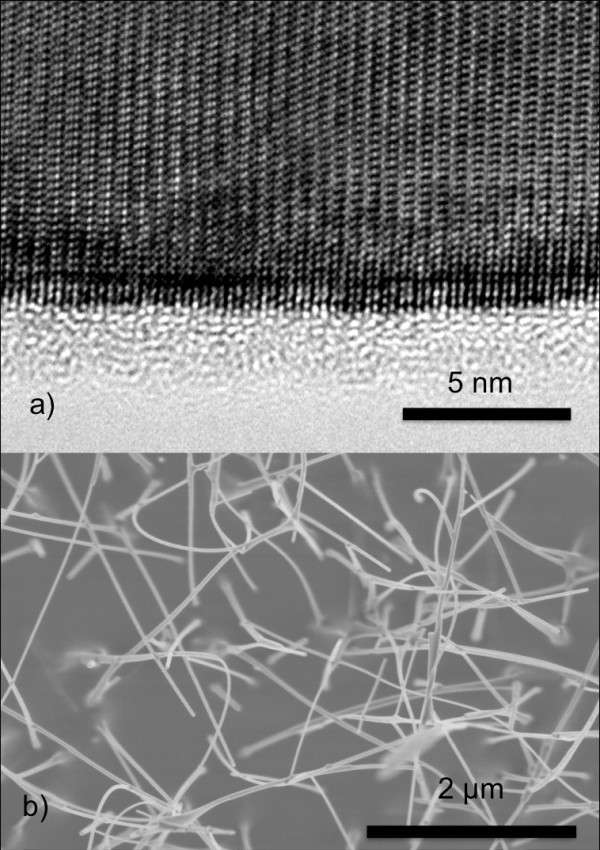
**Bright-field HRTEM and SEM images**. (**a**) Bright-field HRTEM image along the <11-20 > viewing direction of a ZnO nanowire. (**b**) SEM image of ensemble of ZnO nanowires.

As seen in Figure 1a, transmission electron microscopy (TEM) analysis shows that the NWs are single crystalline ZnO with a wurtzite structure. The NW axis is oriented along the [0001] direction, and the structure is free from extended defects. The high-resolution TEM images evidence well-defined lateral sidewalls, parallel to the wire growth direction. No impurities or precipitates have been detected within the accuracy of the energy dispersive X-ray spectroscopy performed in the TEM. The NW morphology was observed by scanning electron microscopy (SEM). A typical top-view SEM image of the as-grown NW ensemble is shown in Figure 1b. The SEM analyses show that the NWs do not have a specific direction with respect to the substrate. The diameter is dispersed in the range of 20 to 100 nm and the length is in the range of 500 nm to 5 μm.

## Results and discussions

For μ-PL studies, single NWs were detached by ultrasound bath from their substrates and dispersed in ethanol on Si substrates patterned with alignment marks. The surface density of NWs is controlled by dispersion in the range of 1 to 5 × 10^6 ^NWs/cm^2^, which is low enough to avoid simultaneous optical excitation of several wires with different orientations. The dispersed NWs do not show any bending and are free of strain. Polarization-resolved μ-PL experiments have been performed in the temperature interval of 4.2 to 300 K. The samples were cooled down in a continuous-flow liquid He cryostat and excited by means of a frequency-doubled continuous-wave Ar++ ion laser at 244 nm. The laser was focused on the substrate surface in a spot with a diameter of 3 μm by means of a UV microscope objective with 0.4 numerical aperture. The excitation power was set in the range of 10 to 50 μW. The sample was imaged through a UV-sensitive camera in order to visualize the luminescence spot and to locate the NW with respect to the alignment marks. μ-PL spectra were measured using a Jobin Yvon HR460 spectrometer (Horiba Ltd., Tokyo, Japan) with a 600- or 1,800-grooves/mm grating and a charge-coupled device camera. The energy resolution of the setup during these experiments is around 1 meV. In order to analyze the polarization of the single NW emission, a linear polarizer was placed at the entrance of the spectrometer. For each individual NW, a series of spectra was collected at different angles of the polarizer axis, which was varied over the whole interval 0° to 360° with a 15° step. The orientation of the NW with respect to the polarizer axis, as well as its isolation from other dispersed nanowires, has been assessed by SEM measurements performed after the optical characterization. The experiment was carried out on ten NWs, yielding a good reproducibility.

Using the alignment marks as a reference frame, we identified the polarizer angles corresponding to π- (light with the electric field *E *_┴ _*c*-axis) and σ- (*E *// *c*-axis) polarizations for each wire. The polarization ratio is defined as:

(1) $$P=\left(I_{\pi }-I_{\sigma }\right)∕\left(I_{\pi }+I_{\sigma }\right)$$

where *I*_π _and *I*_σ _are the integrals of the PL intensity for the π and σ polarizations, respectively.

The polarization of the NBE emission is related to the selection rules for the excitonic transitions, which can be deduced from the k·p theory [19]. The polarization ratio of the three exciton types in strain-free crystal can be expressed as a function of the band parameters of ZnO. The interband momentum-matrix elements $M_{\sigma ,\pi }^{2}$ proportional to the PL intensity for σ and π polarization are reported in the Table 1. It shows that the *X*_A _exciton (formed by an electron bound to a heavy hole) is purely polarized perpendicular to the *c*-axis due to the selection rules in wurtzite crystal. The *X*_B _exciton (an electron bound to a light hole) is strongly polarized perpendicular to the *c*-axis, whereas the *X*_C _exciton (an electron bound to a split-off hole) is strongly polarized parallel to the *c*-axis. The *X*_C _exciton has a much higher energy than the *X*_A _one (energy difference Δ*E*_CA _between the *X*_C _and *X*_A _is 48 meV). Therefore, the NBE photoluminescence is dominated by the lower-energy *X*_A _exciton and, in consequence, is expected to be strongly polarized perpendicular to the *c*-axis even at room temperature (*k*_B_*T *≈ 25 meV < Δ*E*_CA_). It should be noted that many-particle processes can potentially influence the emission polarization. In the polarization analyses, we approximate the NBE emission as originating solely from the *X*_A _bound and free excitons and we neglect the effect of phonon replicas, which are one order of magnitude weaker than the main peak and which could not be detected in single nanowire spectra.

**Table 1:** Normalized interband squared momentum-matrix element $M_{\sigma ,\pi }^{2}$

Exciton type	*E *// *c*-axis [ZnO value]	*E *_┴ _*c*-axis [ZnO value]
*X*_A_	0	0.5
*X*_B_	0.0057	0.4965
*X*_C_	0.9929	0.035

For polarization along the *c*-axis and perpendicular to the *c*-axis for excitons *X*_A_, *X*B, and *X*_C _for ZnO crystal.

**Figure 2:**
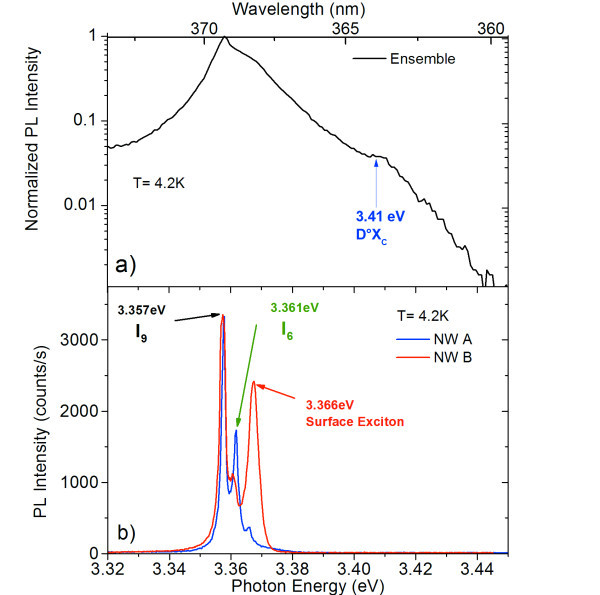
**PL and μPL spectra**. (**a**) PL spectrum of the NW ensemble collected at 4.2 K. (**b**) μPL spectra of two single nanowires (blue and red) collected at 4.2 K with a spectral resolution of 800 μeV.

The PL spectrum of the NW ensemble collected at 4.2 K is reported in Figure 2a. It presents a broad NBE emission peaked at 3.357 eV consisting of different contributions from the bound states of the *X*_A _exciton and possibly a contribution of *X*_B _exciton, which cannot be separately resolved. In addition, a weak shoulder is observed at high energy (3.41 eV) which is related to the *X*_C _exciton. The μ-PL spectra of two single NWs recorded with a spectral resolution of 800 μeV are reported in Figure 2b. All spectra exhibit three narrow peaks with linewidth as low as 1.5 meV. These peaks can be attributed to the different bound states of *X*_A _exciton. The two predominant peaks at 3.357 and 3.361 eV are attributed to the *I*_9 _and *I*_6 _lines related to the neutral donor-bound exciton *D*°*X*_A_, respectively, bound to Al and In [25]. Alternatively, as studied by Meyer et al. [26], theses lines could be related to the neutral donor-bound exciton *D*°*X*_A _and *D*°*X*_B_. The latter interpretation is less probable since the relative intensity of the peaks does not match with the expected population of the corresponding excitonic states at low temperature. In the following analyses, we use the first attribution; however, the conclusions also remain valid for the second one. At higher energy (3.366 eV), we notice a third peak related to a surface bound *X*_A _exciton [27,28]. The intensity of this peak varies from wire to wire due to the NW size dispersion.

**Figure 3:**
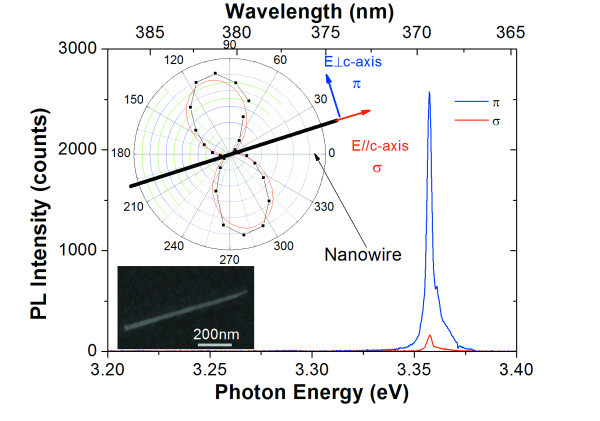
**μ-PL spectra of a single nanowire on carbon-formvar membrane**. Collected at 4.2 K for *E *// *c *and *E *_┴ _*c *with a spectral resolution of 1.5 meV. The polarization diagram and SEM image of a studied nanowire lying on the substrate are reported in the inset.

Typical μ-PL spectra recorded at *T *= 4.2 K for σ- and π-polarizations are reported in Figure 3. By changing the polarization from σ to π, we observe that the spectral shape remains the same within the experimental accuracy, but the PL intensity integrated on the entire spectrum varies of about a factor of 12. This large contrast corresponds to a polarization ratio as high as 0.85. The statistics over ten NWs yields an average polarization ratio of 0.84 with a standard deviation of 0.05. The dependence of the PL intensity on the angle of polarization can be well fitted by a cosine-squared law *I *≈ cos^2^(π/2 - *θ*), where *θ *is the angle between the analyzer and the NW axis determined from the SEM analyses. The maximum luminescence intensity is obtained when the analyzer is perpendicular to the *c*-axis of the NW (π-polarization). From the polarization selection rules, an even higher polarization ratio of 0.98 is expected. The difference between the experimental observation and the theoretical prediction can possibly be explained by a partial depolarization due to the diffraction from the NW of the luminescence exiting the NW extremities. In addition, the dielectric contrast between the NW and its environment, and the elongated shape of ZnO NWs with small diameter (<80 nm), should favor the emission of light polarized parallel to the NW axis [20,21]. However, this effect cannot compete with the high anisotropy of the emission polarization. The polarization of the ZnO luminescence perpendicular to the NW axis is dictated by the excitonic selection rules.

**Figure 4:**
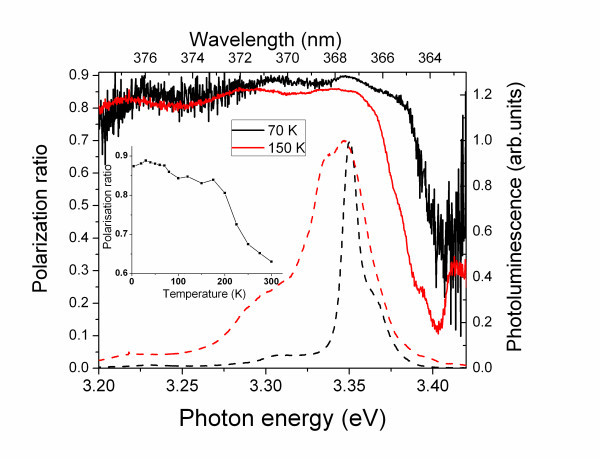
**Polarization ratio of the PL emission from one of the analyzed nanowires**. As a function of the photon energy for temperatures *T *= 70 K (full black curve) and 150 K (full red curve). The normalized PL spectra recorded at *T *= 70 K (dashed black curve) and 100 K (dashed red curve) are reported as reference. The inset reports the polarization ratio of the whole spectrum as a function of temperature.

The temperature dependence of the polarization ratio integrated on the whole spectrum is reported in the inset to the Figure 4. The polarization ratio decreases when the temperature increases from *P *= 0.85 at *T *= 4 K to *P *= 0.63 at *T *= 300 K. This effect is due to the progressive thermal activation of higher energy excitons, in particular of the *X*_C _having a different symmetry. However, the *X*_C _population remains weak even at room temperature, which explains a high polarization ratio (above 0.63) over the whole interval *T *= 4 to 300 K.

It is difficult to observe the gradual activation of the *X*_C _emission directly from the μ-PL spectra because of the very weak signal associated with this transition. However, the *X*_C _luminescence can be evidenced by analyzing the energy-dependent polarization ratio *P*(*E*):

(2) $$P\left(E\right)=\left(I{\left(E\right)}_{\pi }-I{\left(E\right)}_{\sigma }\right)∕\left(I{\left(E\right)}_{\pi }+I{\left(E\right)}_{\sigma }\right)$$

at different temperatures, where *I*(*E*)_*p *_is the PL intensity at energy *E *in the *p *polarization. Figure 4 reports the *P*(*E*) for 70 and 150 K. (The temperature range is restricted within 70 to 150 K due to the extremely low signal above 3.38 eV at low temperatures and to the decrease of the overall luminescence intensity at high temperature). For the energy region between 3.28 and 3.38 eV, the signal arises from the *X*_A _and thermally activated *X*_B _excitonic transitions. Therefore, the polarization ratio remains high (>0.85) in this interval and is nearly temperature independent. At higher energy, around 3.41 eV, the polarization ratio decreases in correspondence of the emission of the *X*_C _exciton. It should be noted that in spite of the weak signal in the spectral range corresponding to the *X*_C _emission, the signal-to-noise ratio is about 20 at 3.40 eV. Therefore, the maximum possible error on the polarization ratio induced by the noise is less than 0.1. With increasing temperature, the dip in the *P*(*E*) dependence is amplified and progressively shifts towards lower energy. This behavior reflects the progressive thermal activation of the *X*_C _excitonic emission and the ZnO bandgap reduction described by the Varshni law [29].

## Conclusions

In conclusion, we have studied the optical properties of ZnO nanowires grown by evaporation technique. Nanowires have defect-free single crystalline structure as shown by high-resolutions TEM (HRTEM) analysis. The nanowires are characterized by an intense photoluminescence with a spectral broadening below 2 meV. We have investigated the polarization dependence of the near-band-edge photoluminescence in ZnO strain-free nanowires. They exhibit a polarization ratio as high as 0.84. We show that these observations are consistent with the k·p theory and with the exciton selection rules. In particular, the weak dependence of the integrated polarization ratio *P *is a consequence of the large energy difference between *X*_A _and *X*_C _excitons. However, the analysis of the energy-resolved polarization ratio *P*(*E*) at different temperatures allows for the observation of the progressive activation of the *X*_C _exciton.

## Abbreviations

NBE: near band edge; NW: nanowire; PL: photoluminescence; μ-PL: microphotoluminescence; SEM: scanning electron microscopy; TEM: transmission electron microscopy.

## Competing interests

The authors declare that they have no competing interests.

## Authors' contributions

GJ carried out the μ-PL measurements and data analysis, performed k·p analysis, and wrote the manuscript. LR and ADLB participated in the μ-PL measurements. LR, MT, and FHJ participated in the data analysis and to the interpretation of the results. CB and EC grew the sample. MF performed the TEM analysis. All authors read and approved the final manuscript.

## References

[B1] Wan Q Li QH Chen YJ Wang TH He XL Li JP Lin CL Fabrication and ethanol sensing characteristics of ZnO nanowire gas sensors Applied Physics Letters 2004 84 3654 10.1063/1.1738932

[B2] Baratto C Todros S Faglia G Comini E Sberveglieri G Lettieri S Santamaria L Maddalena P Luminescence response of ZnO nanowires to gas adsorption Sensors and Actuators B: Chemical 2009 140 461 466 10.1016/j.snb.2009.05.018

[B3] Comini E Baratto C Faglia G Feronni M Vomiero A Sberveglieri G Quasi-one dimensional metal oxide semiconductors: preparation, characterization and application as chemical sensors Progress in Materials Science 2009 54 1 67 10.1016/j.pmatsci.2008.06.003

[B4] Xu S Xu C Liu Y Hu Y Yang R Yang Q Ryou JH Kim HJ Lochner Z Choi S Dupuis R Wang ZL Ordered nanowire array blue/near-UV light emitting diodes Advanced Materials (Deerfield Beach, Fla) 2010 0245 1 5 10.1002/adma.201002134 20862713

[B5] Huang MH Mao S Feick H Yan H Wu Y Kind H Weber E Russo R Yang P Room-temperature ultraviolet nanowire nanolasers Science 2001 292 1897 1899 10.1126/science.1060367 11397941

[B6] Soci C Zhang A Xiang B Dayeh A Aplin DPR Park J Bao XY Lo YH Wang D ZnO nanowire UV photodetectors with high internal gain Nano Letters 2007 7 1003 1009 10.1021/nl070111x 17358092

[B7] Fan Z Chang P-chun Lu JG Walter EC Penner RM Lin CH Lee HP Photoluminescence and polarized photodetection of single ZnO nanowires Applied Physics Letters 2004 85 6128 10.1063/1.1841453

[B8] Hu JQ Bando Y Growth and optical properties of single-crystal tubular ZnO whiskers Applied Physics Letters 2003 82 1401 10.1063/1.1558899

[B9] Vanheusden K Warren WL Seager CH Tallant DR Voigt JA Gnade BE Mechanisms behind green photoluminescence in ZnO phosphor powders Journal of Applied Physics 1996 79 7983 10.1063/1.362349

[B10] Studenikin SA Cocivera M Time-resolved luminescence and photoconductivity of polycrystalline ZnO films Journal of Applied Physics 2002 91 5060 10.1063/1.1461890

[B11] Nam YS Lee SW Baek KS Chang SK Song JH Song JH Han SK Hong SK Yao T Anisotropic optical properties of free and bound excitons in highly strained A-plane ZnO investigated with polarized photoreflectance and photoluminescence spectroscopy Applied Physics Letters 2008 92 201907 10.1063/1.2930683

[B12] Matsui H Tabata H In-plane anisotropy of polarized photoluminescence in M-plane (1010) ZnO and MgZnO/ZnO multiple quantum wells Applied Physics Letters 2009 94 161907 10.1063/1.3124243

[B13] Thomas DG The exciton spectrum of zinc oxide Journal of Physics and Chemistry of Solids 1960 15 86 96 10.1016/0022-3697(60)90104-9

[B14] Hopfield JJ Fine structure in the optical absorption edge of anisotropic crystals Journal of Physics and Chemistry of Solids 1960 15 97 107 10.1016/0022-3697(60)90105-0

[B15] Birman JL Polarization of fluorescence in CdS and ZnS single crystals Physical Review Letters 1959 2 157 159 10.1103/PhysRevLett.2.157

[B16] Kim B Kim H Park S Kyhm K Cho C Polarization asymmetry and optical modal gain saturation via carrier-photon interaction in ZnO Applied Physics Letters 2010 97 041115 10.1063/1.3473729

[B17] Hsu NE Hung WK Chen YF Origin of defect emission identified by polarized luminescence from aligned ZnO nanorods Journal of Applied Physics 2004 96 4671 10.1063/1.1787905

[B18] Rigutti L Tchernycheva M De Luna Bugallo A Jacopin G Julien FH Furtmayr F Stutzmann M Eickhoff M Songmuang R Fortuna F Photoluminescence polarization properties of single GaN nanowires containing Al_x_Ga1-_*x*_N/GaN quantum discs Physical Review B 2010 81 045411

[B19] Chuang S Chang C k·p method for strained wurtzite semiconductors Physical Review B 1996 54 2491 2504 10.1103/PhysRevB.54.2491 9986096

[B20] Ruda HE Shik A Polarization-sensitive optical phenomena in semiconducting and metallic nanowires Physical Review B 2005 72 115308

[B21] Chen HY Yang YC Lin HW Chang SC Gwo S Polarized photoluminescence from single GaN nanorods: effects of optical confinement Optics Express 2008 16 13465 13475 10.1364/OE.16.013465 18711586

[B22] Chien CT Wu MC Chen CW Yang HH Wu JJ Su WF Lin CS Chen YF Polarization-dependent confocal Raman microscopy of an individual ZnO nanorod Applied Physics Letters 2008 92 223102 10.1063/1.2938701

[B23] Yang YR Yan XH Xiao Y Guo ZH The optical properties of one-dimensional ZnO: a first-principles study Chemical Physics Letters 2007 446 98 102 10.1016/j.cplett.2007.08.004

[B24] Cao G Nanostructures & Nanomaterials: Synthesis, Properties & Applications 2004 London: Imperial College Press21874203

[B25] Meyer BK Alves H Hofmann DM Kriegsies W Forster D Bertram F Christen J Hoffmann A Strassburg M Dworzak M Haboeck Rodina AV Bound exciton and donor-acceptor pair recombinations in ZnO Physica Status Solidi (B) 2004 241 231 260 10.1002/pssb.200301962

[B26] Meyer B Sann J Eisermann S Lautenschlzeger S Wagner M Kaiser M Callsen G Reparaz J Hoffman A Excited state properties of donor bound excitons in ZnO Physical Review B 2010 82 115207

[B27] Grabowska J Meaney A Nanda K Mosnier JP Henry M Duclère JR McGlynn E Surface excitonic emission and quenching effects in ZnO nanowire/nanowall systems: limiting effects on device potential Physical Review B 2005 71 1 7

[B28] Wischmeier L Voss T Rückmann I Gutowski J Mofor A Bakin A Waag A Dynamics of surface-excitonic emission in ZnO nanowires Physical Review B 2006 74 1 9

[B29] Cao B Cai W Zeng H Temperature-dependent shifts of three emission bands for ZnO nanoneedle arrays Applied Physics Letters 2006 88 161101 10.1063/1.2195694

